# Temporal and structural evolution of the Early Palæogene rocks of the Seychelles microcontinent

**DOI:** 10.1038/s41598-017-00248-y

**Published:** 2017-03-14

**Authors:** J. Gregory Shellnutt, Meng-Wan Yeh, Kenshi Suga, Tung-Yi Lee, Hao-Yang Lee, Te-Hsien Lin

**Affiliations:** 1 Department of Earth Sciences, National Taiwan Normal University, 88 Tingzhou Road Section 4, Taipei, 116 Taiwan; 2 Center for General Education, National Taiwan Normal University, 162 Heping East Road Section 1, Taipei, 106 Taiwan; 3 Academia Sinica, Institute of Earth Sciences, 128 Academia Road Section 2, Taipei, 115 Taiwan; 4 Department of Geosciences, National Taiwan University, P.O. Box 13-318, Taipei, 106 Taiwan

## Abstract

The Early Palæogene Silhouette/North Island volcano-plutonic complex was emplaced during the rifting of the Seychelles microcontinent from western India. The complex is thought to have been emplaced during magnetochron C28n. However, the magnetic polarities of the rocks are almost entirely reversed and inconsistent with a normal polarity. In this study we present new *in situ* zircon U/Pb geochronology of the different intrusive facies of the Silhouette/North Island complex in order to address the timing of emplacement and the apparent magnetic polarity dichotomy. The rocks from Silhouette yielded weighted mean ^206^Pb/^238^U ages from 62.4 ± 0.9 Ma to 63.1 ± 0.9 Ma whereas the rocks from North Island yielded slightly younger mean ages between 60.6 ± 0.7 Ma to 61.0 ± 0.8 Ma. The secular latitudinal variation from Silhouette to North Island is consistent with the anticlockwise rotation of the Seychelles microcontinent and the measured polarities. The rocks from Silhouette were emplaced across a polarity cycle (C26r-C27n-C27r) and the rocks from North Island were emplaced entirely within a magnetic reversal (C26r). Moreover, the rocks from North Island and those from the conjugate margin of India are contemporaneous and together mark the culmination of rift-related magmatism.

## Introduction

The rifting of the Seychelles microcontinent from western India is contemporaneous with the eruption of the Deccan Traps and is one of the fastest rift-to-drift transitions associated with flood basalt-rifted margins^1–7^. The Seychelles microcontinent is primarily exposed as a series of islands and islets within the Main Islands of the Republic of Seychelles. The Main Islands are almost entirely composed of Neoproterozoic granitic rocks that formed at an Andean-type margin on the edge of Rodinia^8–11^. However, Silhouette and North Island, the western most Main Islands, are Late Cretaceous to Early Palæogene in age and considered to be petrogenetically related to the Deccan Traps^12–15^.

Paleomagnetic and geological studies on the Silhouette/North Island volcano-plutonic complex and the surrounding regional sedimentary basins indicate that the Seychelles microcontinent rotated anticlockwise after magnetochron C28n and may have acted independently of the Indian and African plates^14, 16^. The rocks from Silhouette and North Island, for the exception of a microgranite sample from eastern Silhouette, have reversed magnetic polarities^14^. The magnetic remanence directions are at odds with the weighted radio-isotopic ages of the rocks that suggest the Silhouette/North Island complex was emplaced during a normal polarity^14^. Moreover it is suggested that the Silhouette/North Island complex marks the culmination of Réunion mantle plume activity associated with the Deccan Traps although younger (60.4 ± 0.6 Ma and 61.8 ± 0.6 Ma) silicic volcanic rocks are known to exist around the Mumbai region of western India^15, 17^.

In this study we present new *in situ* zircon U/Pb ages from the different intrusive facies of Silhouette (fayalite-bearing syenite, fayalite-absent syenite, microgranite) and North Island (diorite and syenite) in order to investigate the dichotomy between the magnetic remanence signatures and the radio-isotopic ages from rocks of the Silhouette/North Island complex. Our new results have significant implications for the timing and emplacement of the Deccan-related rocks in the Seychelles microcontinent as it pertains to the structural implications of the anticlockwise rotation of Seychelles microcontinent.

## Geological Background

**Figure 1:**
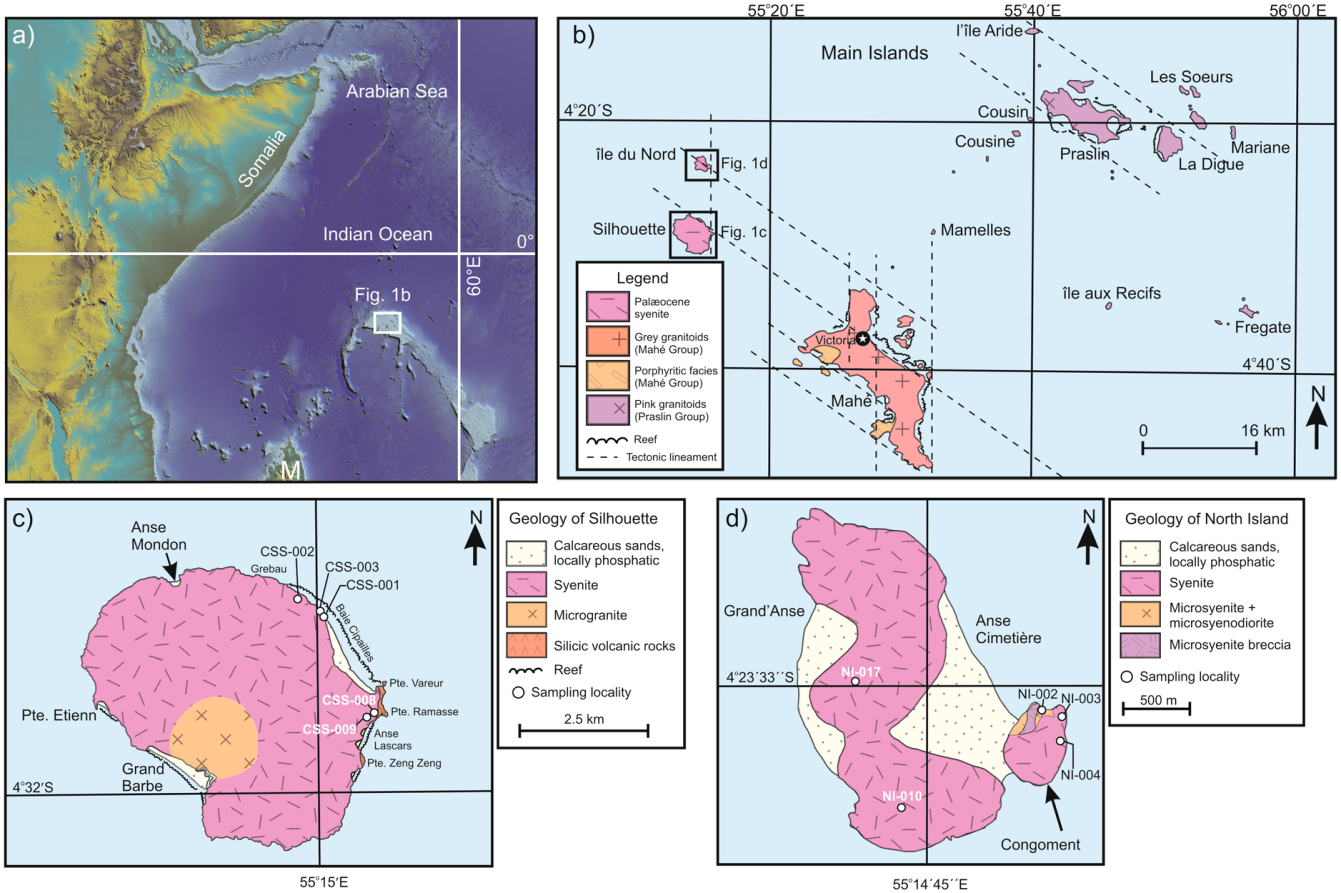
Geological maps of the Seychelles and sampling locations. (**a**) Regional topography of the western Indian Ocean and eastern Africa constructed via Generic Mapping Tools (GMT 4.5.14 http://gmt.soest.hawaii.edu/gmt4/) from Global Relief Model data of ETOPO1, https://www.ngdc.noaa.gov/mgg/global/relief/ETOPO1/data/18. M = Madagascar.﻿ ﻿(**b**) Geological map of the Main Islands of the Seychelles^23^. (**c**) Simplified geological map of Silhouette and the sampling localities for this study^23^. (**d**) Simplified geological map of North Island and the sampling localities for this study^23^.

The Mascarene Plateau is comprised of submerged fragments of ancient continental crust in the western Indian Ocean that covers an area of ~115 000 km^2^ and extends for ~2000 km from north to south^1, 8–11^. The plateau is exposed above the water surface at a few localities (Seychelles, Mauritius, Réunion, Rodrigues and Cargados Carajos Shoals). The Seychelles microcontinent is an elliptical-shaped block located at the northern end of the Mascarene Plateau (Fig. 1a)^18^. The exposed islands and islets of the Seychelles are composed mostly of granitic rocks with subordinate volcanic and mafic intrusive rocks and are ringed by coral reefs^15, 19, 20^.

Geologically there are three groups of islands that correspond to geography, composition and age (Fig. 1b). The granitic rocks of the Mahé Group are grey in colour and range in age from 748 ± 1.2 Ma to 764 ± 1.9 Ma whereas the Praslin Group of granitic rocks are red to pink in colour and are 750 ± 1.8 Ma to 759 ± 2.0 Ma with île aux Recifs ~50 Ma older at 809 ± 1.9 Ma^9, 21^. The Mahé and Praslin groups likely formed at an Andean-type setting on the margin of Rodinia^9, 10^. The youngest islands, Silhouette and North Island, are located at the western edge of the Main Islands and are mainly composed of syenite with a minor amount of mafic to intermediate rocks (Fig. 1b).

Silhouette is located ~20 km NW of Mahé and covers an area of 20.1 km^2^ and is considered to be a ring complex that has a syenitic rim and a granitic core (Fig. 1c). There are two outcroppings of volcanic rocks along the easternmost portion of the island at Pointe Vareur-Pointe Ramasse and Pointe Zeng Zeng (Fig. 1c). The volcanic rocks are trachytic tuffs that contain fragments of other texturally distinct silicic and mafic rocks and were intruded by microgranites along contours suggesting a sill-like emplacement relationship^14^. A total of five samples targeted for radio-isotopic dating were collected from Silhouette. Three fayalite-bearing syenites were collected from the northern end of Baie Cipailles (near Grebau) whereas an additional two fayalite-absent granitic rocks (1 syenite, 1 microgranite) were collected near Pointe Vareur (Fig. 1c). North Island (île du Nord) is ~5 km north of Silhouette and has an area of 2.01 km^2^. The principal rock-type found on North Island is a buff grey syenite however there is a gabbro ‘float’ near the centre of the island and there is a small exposure of darker olivine-biotite-bearing gabbro/diorite to the southwest which forms a portion of the Congoment promontory (Fig. 1d)^15, 19^. In the north, porphyritic microsyenite dykes and dark-coloured veins are also observed trending to the northwest. A total of five samples were collected for radio-isotopic dating. Three samples were collected from Congoment (2 syenites, 1 diorite) whereas the remaining two were collected from the main north-south trending syenite body (Fig. 1d).

## Results

The zircon laser ablation inductively coupled plasma mass spectrometry (LA-ICP-MS) weighted mean ^206^Pb/^238^U results for this study are listed in Table 1. The complete dataset is available in the online supplementary information (Datasets S1 and S2). The zircons from Silhouette and North Island vary in size (50 μm to 450 μm long) and display oscillatory zonation (Figs S1 and S2). Most crystals are euhedral to subhedral with equant to elongate shapes but some crystals have fragmented shapes. The fragmented shapes are probably caused by the mineral separation process. The measured Th/U ratios for all zircons are ≥0.4 and within the range of typical igneous zircons^22^.

**Table 1:** Summary of mean zircon ^206^Pb/^238^U age dates from the Silhouette/North Island complex.

Sample	Island	Latitude (dms)	Longitude (dms)	Material	Age (Ma)
NI-002	North Island	4°23′39″ S	55°15′06″ E	Zircon (diorite)	60.6 ± 0.7
NI-003	North Island	4°23′39″ S	55°15′10″ E	Zircon (syenite)	61.0 ± 0.5
NI-004	North Island	4°23′43″ S	55°15′10″ E	Zircon (syenite)	60.9 ± 0.7
NI-010	North Island	4°23′57″ S	55°14′41″ E	Zircon (syenite)	61.0 ± 0.8
NI-017	North Island	4°23′38″ S	55°14′29″ E	Zircon (syenite)	60.7 ± 0.8
CSS-001	Silhouette	4°28′15″ S	55°14′36″ E	Zircon (syenite)	62.4 ± 0.9
CSS-002	Silhouette	4°28′04″ S	55°14′12″ E	Zircon (syenite)	63.1 ± 0.9
CSS-003B	Silhouette	4°28′14″ S	55°14′34″ E	Zircon (syenite)	62.7 ± 0.7
CSS-008	Silhouette	4°29′20″ S	55°15′19″ E	Zircon (microgranite)	62.5 ± 0.8
CSS-009	Silhouette	4°29′22″ S	55°15′14″ E	Zircon (syenite)	62.4 ± 1.0

The complete dataset can be found in Dataset S1. The geographic coordinates are in degrees, minutes and seconds (dms).

The weighted mean ages of Silhouette range from 62.4 ± 0.9 Ma to 63.1 ± 0.9 Ma (2σ uncertainty). The three fayalite-bearing syenites collected from northern Silhouette have ages (62.4 ± 0.9 Ma, 62.7 ± 0.7 Ma, 63.1 ± 0.9 Ma) indistinguishable within error of the fayalite-absent syenite and microgranite from eastern Silhouette (62.4 ± 1.0 Ma to 62.5 ± 0.8 Ma). Regression of all data points from each sample yield Concordia or intercept ages that are indistinguishable from the weighted mean ages (62.6 ± 0.9 Ma, 62.6 ± 1.1 Ma, 62.7 ± 0.7 Ma, 62.8 ± 0.9 Ma, 63.1 ± 0.9 Ma) (Fig. S3).

The zircons separated from the North Island rocks yielded similar weighted mean (2σ uncertainly) ages (60.6 ± 0.7 Ma, 60.7 ± 0.8, 60.9 ± 0.7, 61.0 ± 0.5, 61.0 ± 0.8) but are consistently younger than the rocks from Silhouette. The Concordia or intercept ages are indistinguishable from the weighted mean ages (60.6 ± 0.9 Ma, 60.7 ± 0.3 Ma, 60.8 ± 0.8 Ma, 60.9 ± 0.9 Ma, 61.1 ± 0.7 Ma) (Fig. S4).

### Temporal evolution of the Silhouette/North Island complex

**Figure 2:**
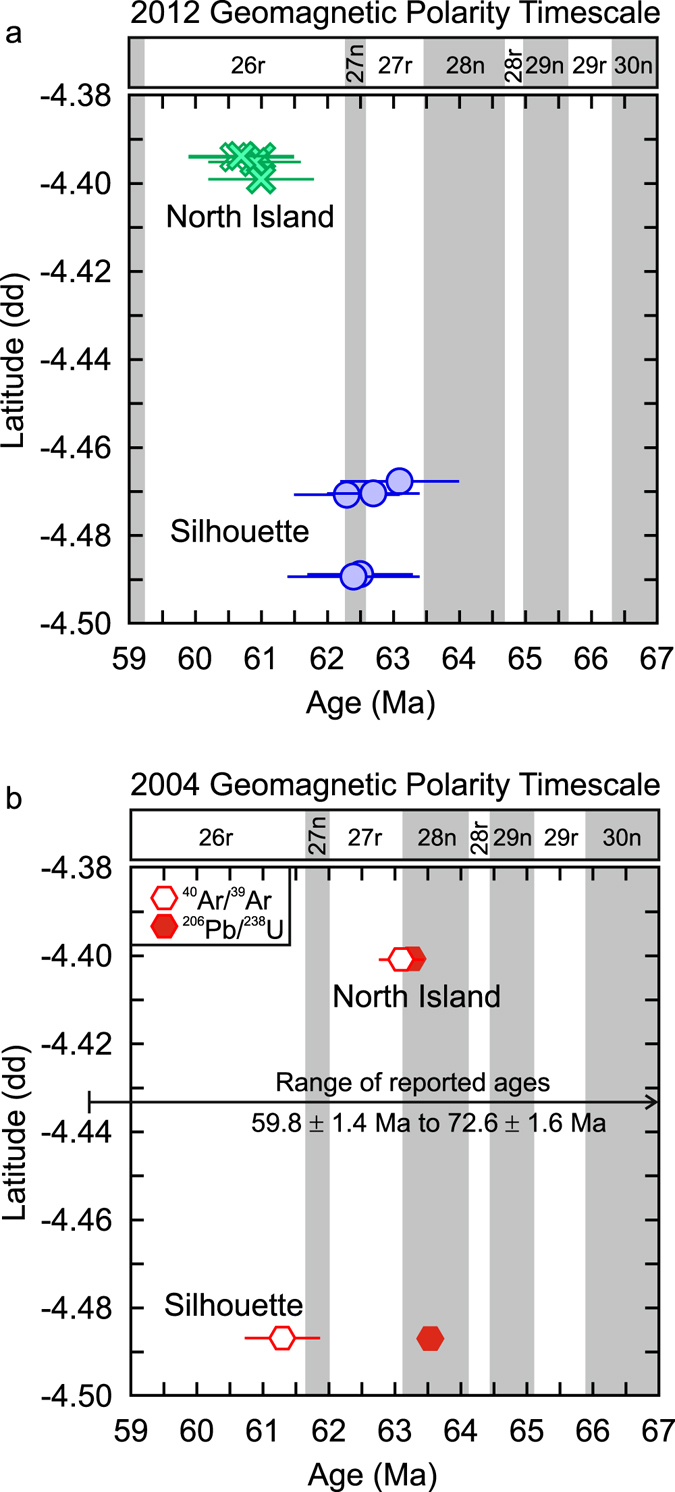
Secular latitudinal variation of the rocks from the Silhouette/North Island complex. (**a**) The zircon U/Pb ages (with uncertainty) are plotted based on their latitude in decimal degrees (dd) and the 2012 geomagnetic polarity timescale^24^. (**b**) The weighted zircon U/Pb ages (with uncertainty)^14^, weighted mineral^40^ Ar/Ar^39^ ages (with uncertainty)^14^ and complete range of previously reported ages of Silhouette and North Island are plotted relative to the 2004 geomagnetic polarity timescale^1, 2, 14, 43^.

The results from this study are within the range of previously reported ages of the Silhouette/North Island complex^1, 2, 14, 23^. However, we are able to identify a distinct spatial-temporal progression from Silhouette to North Island. Figure 2a shows the weighted mean ^206^Pb/^238^U ages with respect to latitude and the 2012 geomagnetic polarity timescale^24^. There is a clear latitudinal secular variation as the ages systematically decrease northward. The rocks from North Island are ~2 million years younger than the rocks from Silhouette. Although there is overlap, it is clear that there is a statistically meaningful age difference between the rocks of the two islands.

The identification of secular variation within the Silhouette/North Island complex has a number of important magmatic and tectonic implications for the Seychelles microcontinent. The age gap between Silhouette and North Island suggests there are at least two periods of magmatism within the complex. The trachytic tuffs and the fayalite-absent syenitic rocks from eastern Silhouette (Pte. Vareur-Pte. Ramasse) likely formed the initial volcanic-hypabyssal complex during the early stages (~63 Ma). The only rocks from Silhouette that were found to contain inherited zircons are located in the eastern part of the island where the microgranite and fayalite-absent syenite were collected^23^. Based on the abundance of xenoliths and micro-xenoliths within the trachytic rocks from eastern Silhouette, it is likely that they erupted violently thereby brecciating the country rocks and allowing them to be incorporated into the tuff. The emplacement of the fayalite-bearing rocks could represent a second period of magmatism as they are texturally distinct from the fayalite-absent syenites. However, the fayalite-bearing and fayalite-absent rocks have similar whole rock and Sr-Nd isotopic compositions and ages thus it is possible that the rocks formed by the same process but that the principal difference between their magmas was volatile (H_2_O, CO_2_, Cl, F) content as fayalite crystallizes under relatively dry conditions^2, 15, 25^. The last period of silicic plutonism (~60 Ma) is represented by North Island. The syenitic rocks from North Island are texturally and mineralogically different from the syenites of Silhouette^15,19^. Moreover the whole rock Sr-Nd-Hf isotopes are subtly different. Although there is overlap, the rocks from the Silhouette have slightly higher ^87^Sr/^86^Sr_i_ (0.7035 to 0.7061) ratios, and lower ε_Nd_(t) (+0.5 to +2.9) and ε_Hf_(t) (+3.8 to +5.2) values than North Island (^87^Sr/^86^Sr_i_ = 0.7036 to 0.7041; ε_Nd_(t) = +1.4 to +3.8; ε_Hf_(t) = +4.6 to +6.2)^2, 15^.

Regionally, the ~3 million year duration of the Silhouette/North Island complex corresponds to the transition between the Deccan (c. 67–63 Ma) and post-Deccan (c. 63–58 Ma) stages of India-Seychelles rifting^3, 26^. The rocks from Silhouette were likely emplaced during the Deccan-stage of rifting just as sea-floor spreading (>63.4 Ma) was initiated whereas the rocks from North Island were emplaced well after sea-floor spreading (<63.4 Ma) was underway. Furthermore, the rocks from North Island are contemporaneous with trachytes (60.4 ± 0.6 Ma and 61.8 ± 0.6 Ma) from western India (Manori and Saki Naka)^4, 17^. Therefore it is likely that only North Island was emplaced during the waning stages of Deccan-related magmatism and together with the trachytes from Manori and Saki Naka mark the culmination of magmatism.

### The magnetic polarity dichotomy

Magnetic polarity measurements of the Silhouette/North Island complex shows that the majority of rocks have reverse magnetic polarity except for one microgranite sample from eastern Silhouette^14^. The magnetic polarity results are inconsistent with the geochronological results produced in the same study (Silhouette ^206^Pb/^238^U = 63.54 ± 0.06 Ma, ^40^Ar/^39^Ar = 61.3 ± 0.6 Ma; North Island ^206^Pb/^238^U = 63.27 ± 0.05 Ma, ^40^Ar/^39^Ar = 63.1 ± 0.34 Ma) as it is suggested the Silhouette/North Island complex was emplaced during a normal polarity magnetochron (C28n). However, the geochronological results from this and previous studies are consistent with the 2012 geomagnetic polarity time scale rather than the 2004 version of the time scale that was used in the study (Fig. 2b)^14, 24^. It is clear that the ages from North Island cluster within magnetochron C26r and are thus compatible with the magnetic polarity results (Fig. 2a). The ages from Silhouette, however, straddle the C26r-C27n-C27r magnetochrons. The precision of the data from this study is insufficient to resolve the short duration of magnetochron C27n (0.296 Ma) but a microgranite collected from eastern Silhouette has a normal polarity and could have been emplaced during that short normal polarity timeframe^14^. Therefore it is possible that the rocks of Silhouette were emplaced across magnetochrons C26r-C27n-C27r (62.221 Ma to 63.494 Ma) and may have either a normal or reverse polarity^14, 24^.

### Anticlockwise rotation of the Seychelles microcontinent and the structural influence on the Silhouette/North Island complex

The locations of Silhouette and North Island appear to be structurally controlled. There are distinct NW-SE and N-S structural lineaments that are evident from the Main Islands morphology and are complementary to regional Bouguer gravity maps (Fig. 1b)^27^. The structural lineaments likely exerted a significant control on the morphology of the Main Islands and it is probably not a coincidence that both Silhouette and North Island are located in close proximity to the junction between two lineaments^27^. Interestingly, the long axis of Silhouette is aligned with a NW-SE lineament whereas the long axis of North Island is aligned with a N-S lineament. The different orientations of the islands, and possibly their locations, suggests that they were emplaced under different stress regimes that reflect the secular evolution of the regional stress field as the Indian Ocean developed^6, 7, 28^.

**Figure 3:**
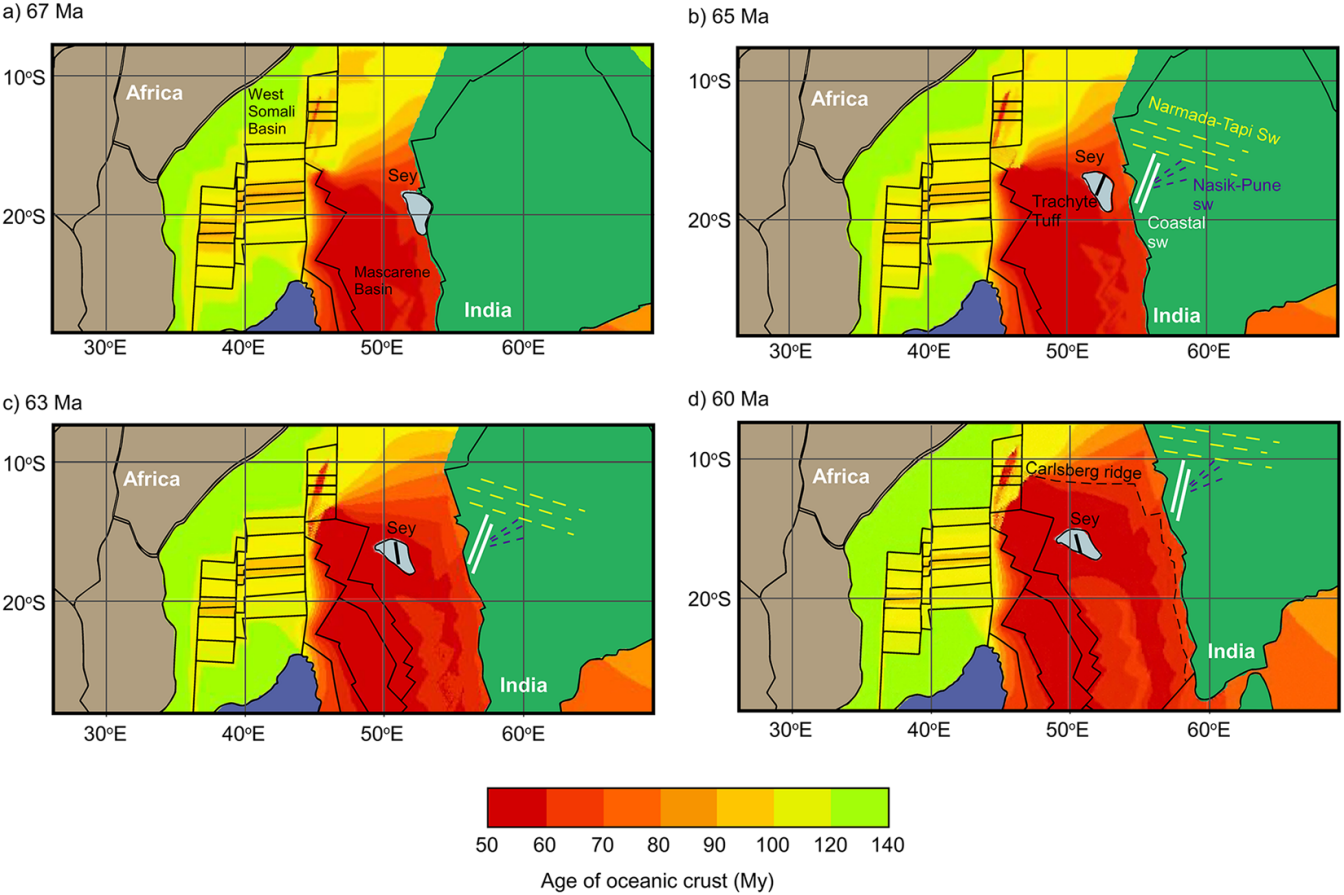
Reconstructed tectonic evolution of Seychelles micro-continent during Early Palæogene via GPlates 1.5 software https://sourceforge.net/projects/gplates/files/gplates/1.5/ and the sample source data provided by Earthbyte for paleotectonic evolution https://www.earthbyte.org/gplates-1-5-software-and-data-sets/, and oceanic crust evolution https://www.earthbyte.org/age-spreading-rates-and-spreading-asymmetry-of-the-worlds-ocean-crust/. (**a**) Paleo-position of the Seychelles microcontinent next to western India at 67 Ma. (**b**) Rifting of the Seychelles from India. The paleo-orientation of trachyte tuff is parallel to the Coastal swarm around 65 Ma. (**c**) Continuous rifting and anticlockwise rotating of Seychelles around 63 Ma. (**d**) Cessation of Seychelles rotation around 60 Ma. The color bar marks the age of oceanic crust. The thin black lines marked the ridges within the West Somali basin and the Mascarene basin.

Paleomagnetic data indicate the Seychelles microcontinent rotated 29° ± 12.9° in an anticlockwise direction after it rifted from India (~64 Ma) to ~60 Ma or possibly later^14^. If the Seychelles microcontinent is rotated back to its original orientation before rifting (66 Ma) and the formation of the first magnetochron in the East Somali basin (C28), the lineaments are oriented NNW-SSE and NNE-SSW. The original position of the Seychelles microcontinent prior 66 Ma has a tight fit with the western Indian margin. By fitting the triangular geometry of the Seychelles into the western Indian margin, a much smoother continental margin with a general N-S alignment can be reconstructed (Fig. 3a). The orientation of the Seychelles microcontinent also agrees with the general N-S trending Late Cretaceous India–Madagascar rift as the Laxmi Basin stopped developing at ~62.5 Ma^28^.

There are a number of dyke swarms emplaced along the western margin of India that are related to the Deccan Traps and include the ENE-WSW striking Narmada-Tapi swarm, the NNE-SSW striking Nasik-Pune swarm and the N-S striking Coastal swarm (Fig. 3b)^29, 30^. The Nasik-Pune and Coastal swarms were emplaced during E-W tensional stress and generally match the orientation of the reoriented Seychelles lineaments^29, 31, 32^. Silhouette was emplaced concurrently (~63 to ~62 Ma) with the dyke swarms during the E-W directed tensional stress as the Seychelles microcontinent rifted from India. It is possible that the initial orientation of the lineaments favoured the emplacement of magmas along the N-S axis when the microcontinent initially rifted and then, after rotation, became the NW-SE lineaments (Fig. 3c). Approximately two million years after Silhouette, North Island (~60 Ma) was emplaced along a N-S lineament after the rotation ceased. The principal plate stress changed from E-W to N-S due to the northward movement of India and the initiation of N-S sea-floor spreading along the Carlsberg Ridge (Fig. 3d). Therefore we suggest the geographic position and orientation of North Island relative to Silhouette is likely due to the structural controls (lineaments, rotation) within the Seychelles microcontinent as it rifted from India and subsequently rotated.

## Conclusions

*In situ* zircon U/Pb age dates of the Early Palæogene rocks from the Seychelles identify a secular variation between Silhouette Island and North Island. The northward decreasing age progression is structurally consistent with the anticlockwise rotation of the Seychelles microcontinent after rifting from western India. Moreover the new ages are in agreement with the magnetic polarities of the rocks suggesting the Silhouette/North Island complex was emplaced over a ~3 million year period beginning during magnetochron C27r and ending during magnetochron 26r. Furthermore, syenites from North Island and trachytes from western India are contemporaneous (~60 Ma) and together mark the culmination of rift-related magmatism.

## Methods

### Geochronology

Zircons were mechanically separated at the Yu-Neng Rock and Mineral Separation Company (Lanfang, Hubei) using a steel jaw crusher, magnetic separation and heavy-liquids. The crystals were linearly mounted on a glass slide covering a diameter of one inch. A mold was placed over the zircons and epoxy was poured over the zircons to ensure transfer of the minerals to the epoxy. Cathodoluminescence (CL) images were taken for selecting suitable positions for spot U/Pb analyses at Institute of Earth Sciences, Academia Sinica. Zircon U/Pb isotopic analyses were performed by the LA-ICPMS technique using an Agilent 7500s ICP-MS and a New Wave 193-nm laser ablation system set up at the Department of Geosciences, National Taiwan University^33^. A spot size of 40 μm with laser repetition rate of 5 Hz was used. Calibration was performed by using the zircon standards GJ-1, 91500 and Plešovice for data quality control^34–36^. Measured U-Th-Pb isotopic ratios were calculated using the GLITTER 4.4.4. software^37^. Common lead was directly corrected using the common lead correction method^38, 39^, and the weighted mean U/Pb ages and concordia plots were carried out using Isoplot v. 4.1^40^. The full table of zircon U/Pb results can be found in Dataset 1 and Dataset 2.

### Plate Reconstruction

GPlates 1.5 software and the sample source data provided: http://www.earthbyte.org/Resources/earthbyte_gplates_1.5_data_sources.html was used to reconstruct paleogeography evolution of Seychelles during the Early Palæogene. A global rotation model was used to reconstruct the paleogeography of India, Africa and Madagascar using Madagascar as the anchor plate^41^. The age of oceanic crust is reconstructed following the Global Present Day Age grid^42^. The evolution of Seychelles microcontinent is modified according to our new age results and previously models^3, 4, 14^.

## Electronic supplementary material

Dataset 1
