# Supplementary material for: Temporal and structural evolution of the Early Palæogene rocks of the Seychelles microcontinent
Source: Sci Rep. 2017 Mar 14;7:179. doi: 10.1038/s41598-017-00248-y (PMC5428008; doi:10.1038/s41598-017-00248-y)
Supplement: Supplementary file 1 — Dataset 1 [file 41598_2017_248_MOESM1_ESM.doc]

# Supplementary Information

# Temporal and structural evolution of the Early Palæogene rocks of the Seychelles microcontinent

J. Gregory Shellnutt^1^, Meng-Wan Yeh^1,2^, Kenshi Suga^3^, Tung-Yi Lee^1^, Hao-Yang Lee^3^ & Te-Hsien Lin^4^

*^1^National Taiwan Normal University, Department of Earth Sciences, 88 Tingzhou Road Section 4, Taipei 116, Taiwan*

*^2^Center for General Education, National Taiwan Normal University, 162 Heping East Road Section 1, Taipei 106, Taiwan*

*^3^Academia Sinica, Institute of Earth Sciences, 128 Academia Road Section 2, Taipei 115, Taiwan*

*^4^Department of Geosciences, National Taiwan University, P.O. Box 13-318, Taipei 106, Taiwan*

*Correspondence and requests for materials should be addressed to J.G.S. (email:jgshelln@ntnu.edu.tw)

**Supplementary Dataset S1**

**Dataset S1.** Zircon LA-ICP-MS geochronology results from Silhouette

| *Analysis* | *Th/U* | *^207^Pb/^206^Pb* | ± 1σ | *^207^Pb/^235^U* | ± 1σ | *^206^Pb/^238^U* | ± 1σ | *Error Correlation* | *^207^Pb/^235^U*  *Age (Ma)* | ± 1σ | *^206^Pb/^238^U*  *Age (Ma)* | ± 1σ |
| --- | --- | --- | --- | --- | --- | --- | --- | --- | --- | --- | --- | --- |
| ***CSS-001 Fayalite syenite*** | |  |  |  |  |  |  |  |  |  |  |  |
| CSS-001-1 | 0.58 | 0.0376 | 0.0050 | 0.0498 | 0.0066 | 0.0096 | 0.0003 | 0.2110 | 49 | 6 | 62 | 2 |
| CSS-001-2 | 0.58 | 0.0433 | 0.0033 | 0.0573 | 0.0045 | 0.0096 | 0.0003 | 0.3485 | 57 | 4 | 62 | 2 |
| CSS-001-3 | 0.55 | 0.0448 | 0.0058 | 0.0586 | 0.0076 | 0.0095 | 0.0003 | 0.2278 | 58 | 7 | 61 | 2 |
| CSS-001-4 | 1.02 | 0.0402 | 0.0020 | 0.0523 | 0.0027 | 0.0094 | 0.0003 | 0.5206 | 52 | 3 | 61 | 2 |
| CSS-001-5 | 0.47 | 0.0512 | 0.0060 | 0.0688 | 0.0081 | 0.0098 | 0.0003 | 0.2519 | 68 | 8 | 63 | 2 |
| CSS-001-6 | 0.59 | 0.0359 | 0.0048 | 0.0475 | 0.0064 | 0.0096 | 0.0003 | 0.2076 | 47 | 6 | 62 | 2 |
| CSS-001-7 | 0.60 | 0.0466 | 0.0048 | 0.0600 | 0.0062 | 0.0093 | 0.0002 | 0.2488 | 59 | 6 | 60 | 2 |
| CSS-001-8 | 0.83 | 0.0402 | 0.0024 | 0.0535 | 0.0032 | 0.0096 | 0.0002 | 0.4024 | 53 | 3 | 62 | 1 |
| CSS-001-9 | 0.67 | 0.0430 | 0.0038 | 0.0594 | 0.0052 | 0.0100 | 0.0003 | 0.2854 | 59 | 5 | 64 | 2 |
| CSS-001-10 | 0.58 | 0.0427 | 0.0044 | 0.0575 | 0.0060 | 0.0098 | 0.0003 | 0.2465 | 57 | 6 | 63 | 2 |
| CSS-001-11 | 0.89 | 0.0496 | 0.0032 | 0.0666 | 0.0043 | 0.0097 | 0.0002 | 0.3682 | 65 | 4 | 62 | 1 |
| CSS-001-12 | 0.61 | 0.0336 | 0.0039 | 0.0455 | 0.0053 | 0.0098 | 0.0003 | 0.2186 | 45 | 5 | 63 | 2 |
| CSS-001-13 | 0.90 | 0.0430 | 0.0019 | 0.0576 | 0.0026 | 0.0097 | 0.0002 | 0.5450 | 57 | 3 | 62 | 2 |
| CSS-001-14 | 0.47 | 0.0468 | 0.0048 | 0.0617 | 0.0064 | 0.0096 | 0.0003 | 0.2532 | 61 | 6 | 61 | 2 |
| CSS-001-15 | 0.82 | 0.0405 | 0.0037 | 0.0533 | 0.0048 | 0.0096 | 0.0002 | 0.2766 | 53 | 5 | 61 | 2 |
| CSS-001-16 | 1.02 | 0.0416 | 0.0023 | 0.0558 | 0.0031 | 0.0097 | 0.0002 | 0.4482 | 55 | 3 | 62 | 2 |
| CSS-001-17 | 0.67 | 0.0426 | 0.0023 | 0.0581 | 0.0031 | 0.0099 | 0.0002 | 0.4486 | 57 | 3 | 63 | 2 |
| CSS-001-18 | 0.65 | 0.0630 | 0.0038 | 0.0876 | 0.0053 | 0.0101 | 0.0003 | 0.4248 | 85 | 5 | 65 | 2 |
| CSS-001-19 | 0.55 | 0.0391 | 0.0034 | 0.0538 | 0.0047 | 0.0100 | 0.0003 | 0.2858 | 53 | 5 | 64 | 2 |
| CSS-001-20 | 0.68 | 0.0490 | 0.0027 | 0.0672 | 0.0038 | 0.0099 | 0.0002 | 0.4294 | 66 | 4 | 64 | 2 |
|  |  |  |  |  |  |  |  |  |  |  |  |  |
| ***CSS-002 Fayalite syenite*** | |  |  |  |  |  |  |  |  |  |  |  |
| CSS-002-1 | 0.65 | 0.0312 | 0.0052 | 0.0410 | 0.0068 | 0.0095 | 0.0003 | 0.1697 | 41 | 7 | 61 | 2 |
| CSS-002-2 | 1.07 | 0.0264 | 0.0028 | 0.0357 | 0.0038 | 0.0098 | 0.0003 | 0.2575 | 36 | 4 | 63 | 2 |
| CSS-002-3 | 0.85 | 0.0430 | 0.0027 | 0.0581 | 0.0037 | 0.0098 | 0.0003 | 0.4220 | 57 | 4 | 63 | 2 |
| CSS-002-4 | 0.63 | 0.0409 | 0.0029 | 0.0556 | 0.0040 | 0.0099 | 0.0003 | 0.3836 | 55 | 4 | 63 | 2 |
| CSS-002-5 | 0.71 | 0.0498 | 0.0041 | 0.0673 | 0.0056 | 0.0098 | 0.0003 | 0.3294 | 66 | 5 | 63 | 2 |
| CSS-002-7 | 0.58 | 0.0517 | 0.0071 | 0.0699 | 0.0096 | 0.0098 | 0.0003 | 0.2160 | 69 | 9 | 63 | 2 |
| CSS-002-8 | 0.75 | 0.0494 | 0.0056 | 0.0646 | 0.0073 | 0.0095 | 0.0003 | 0.2512 | 64 | 7 | 61 | 2 |
| CSS-002-9 | 0.60 | 0.0421 | 0.0091 | 0.0563 | 0.0122 | 0.0097 | 0.0003 | 0.1477 | 56 | 12 | 62 | 2 |
| CSS-002-10 | 1.02 | 0.0428 | 0.0042 | 0.0567 | 0.0056 | 0.0096 | 0.0003 | 0.2826 | 56 | 5 | 62 | 2 |
| CSS-002-11 | 0.42 | 0.0682 | 0.0109 | 0.0965 | 0.0154 | 0.0103 | 0.0003 | 0.2074 | 94 | 14 | 66 | 2 |
| CSS-002-12 | 0.60 | 0.0423 | 0.0053 | 0.0569 | 0.0072 | 0.0098 | 0.0003 | 0.2282 | 56 | 7 | 63 | 2 |
| CSS-002-13 | 0.68 | 0.0383 | 0.0055 | 0.0510 | 0.0073 | 0.0097 | 0.0003 | 0.2029 | 50 | 7 | 62 | 2 |
| CSS-002-14 | 0.94 | 0.0455 | 0.0025 | 0.0608 | 0.0035 | 0.0097 | 0.0003 | 0.4704 | 60 | 3 | 62 | 2 |
| CSS-002-16 | 0.79 | 0.0444 | 0.0034 | 0.0611 | 0.0047 | 0.0100 | 0.0003 | 0.3547 | 60 | 4 | 64 | 2 |
| CSS-002-17 | 0.57 | 0.0569 | 0.0085 | 0.0783 | 0.0116 | 0.0100 | 0.0003 | 0.2090 | 77 | 11 | 64 | 2 |
| CSS-002-18 | 0.45 | 0.0407 | 0.0025 | 0.0555 | 0.0034 | 0.0099 | 0.0003 | 0.4407 | 55 | 3 | 63 | 2 |
| CSS-002-19 | 0.74 | 0.0653 | 0.0058 | 0.0906 | 0.0080 | 0.0101 | 0.0003 | 0.3253 | 88 | 7 | 65 | 2 |
| CSS-002-20 | 0.41 | 0.0594 | 0.0152 | 0.0844 | 0.0215 | 0.0103 | 0.0004 | 0.1489 | 82 | 20 | 66 | 2 |
|  |  |  |  |  |  |  |  |  |  |  |  |  |
| ***CSS-003B Fayalite syenite*** | |  |  |  |  |  |  |  |  |  |  |  |
| CSS-003B-1 | 0.67 | 0.0426 | 0.0015 | 0.0590 | 0.0021 | 0.0100 | 0.0002 | 0.6751 | 58 | 2 | 64 | 2 |
| CSS-003B-2 | 0.67 | 0.0472 | 0.0017 | 0.0633 | 0.0024 | 0.0097 | 0.0002 | 0.6336 | 62 | 2 | 62 | 1 |
| CSS-003B-3 | 0.42 | 0.1038 | 0.0065 | 0.1409 | 0.0086 | 0.0098 | 0.0003 | 0.5013 | 72 | 9 | 59 | 2 |
| CSS-003B-4 | 0.70 | 0.0430 | 0.0018 | 0.0578 | 0.0025 | 0.0098 | 0.0002 | 0.5563 | 57 | 2 | 63 | 1 |
| CSS-003B-5 | 1.14 | 0.0433 | 0.0017 | 0.0595 | 0.0024 | 0.0100 | 0.0002 | 0.5987 | 59 | 2 | 64 | 2 |
| CSS-003B-6 | 0.60 | 0.0438 | 0.0031 | 0.0573 | 0.0040 | 0.0095 | 0.0002 | 0.3595 | 57 | 4 | 61 | 2 |
| CSS-003B-7 | 0.74 | 0.0424 | 0.0017 | 0.0576 | 0.0023 | 0.0099 | 0.0002 | 0.6103 | 57 | 2 | 63 | 2 |
| CSS-003B-8 | 0.69 | 0.0426 | 0.0022 | 0.0578 | 0.0030 | 0.0099 | 0.0002 | 0.4728 | 57 | 3 | 63 | 2 |
| CSS-003B-9 | 0.90 | 0.0468 | 0.0017 | 0.0627 | 0.0023 | 0.0097 | 0.0002 | 0.6373 | 62 | 2 | 62 | 1 |
| CSS-003B-10 | 0.66 | 0.0431 | 0.0018 | 0.0593 | 0.0025 | 0.0100 | 0.0002 | 0.5617 | 59 | 2 | 64 | 2 |
| CSS-003B-11 | 0.67 | 0.0438 | 0.0017 | 0.0588 | 0.0023 | 0.0097 | 0.0002 | 0.6087 | 58 | 2 | 62 | 1 |
| CSS-003B-12 | 0.63 | 0.0472 | 0.0022 | 0.0631 | 0.0030 | 0.0097 | 0.0002 | 0.5291 | 62 | 3 | 62 | 2 |
| CSS-003B-13 | 0.63 | 0.0440 | 0.0018 | 0.0607 | 0.0026 | 0.0100 | 0.0002 | 0.5622 | 60 | 2 | 64 | 2 |
| CSS-003B-14 | 0.67 | 0.0441 | 0.0017 | 0.0608 | 0.0025 | 0.0100 | 0.0002 | 0.5880 | 60 | 2 | 64 | 2 |
| CSS-003B-15 | 0.63 | 0.0451 | 0.0015 | 0.0622 | 0.0022 | 0.0100 | 0.0002 | 0.6689 | 61 | 2 | 64 | 2 |
| CSS-003B-16 | 0.61 | 0.0387 | 0.0020 | 0.0536 | 0.0029 | 0.0101 | 0.0003 | 0.4661 | 53 | 3 | 65 | 2 |
| CSS-003B-17 | 0.95 | 0.0428 | 0.0027 | 0.0585 | 0.0037 | 0.0099 | 0.0003 | 0.4012 | 58 | 4 | 64 | 2 |
| CSS-003B-18 | 0.70 | 0.0516 | 0.0025 | 0.0713 | 0.0035 | 0.0100 | 0.0003 | 0.5110 | 70 | 3 | 64 | 2 |
| CSS-003B-19 | 0.47 | 0.0427 | 0.0023 | 0.0593 | 0.0032 | 0.0101 | 0.0003 | 0.4649 | 58 | 3 | 65 | 2 |
| CSS-003B-20 | 0.77 | 0.0454 | 0.0023 | 0.0597 | 0.0031 | 0.0096 | 0.0002 | 0.4905 | 59 | 3 | 61 | 2 |
|  |  |  |  |  |  |  |  |  |  |  |  |  |
| ***CSS-008 Microgranite*** | | |  |  |  |  |  |  |  |  |  |  |
| CSS-008-1 | 1.60 | 0.0429 | 0.0017 | 0.0585 | 0.0025 | 0.0099 | 0.0002 | 0.5788 | 58 | 2 | 64 | 2 |
| CSS-008-2 | 1.09 | 0.0478 | 0.0026 | 0.0649 | 0.0035 | 0.0098 | 0.0002 | 0.4659 | 64 | 3 | 63 | 2 |
| CSS-008-3 | 0.91 | 0.0556 | 0.0041 | 0.0762 | 0.0056 | 0.0099 | 0.0003 | 0.3546 | 61 | 7 | 63 | 2 |
| CSS-008-5 | 0.78 | 0.0898 | 0.0050 | 0.1233 | 0.0069 | 0.0100 | 0.0003 | 0.4646 | 62 | 9 | 61 | 2 |
| CSS-008-6 | 0.82 | 0.0425 | 0.0036 | 0.0591 | 0.0050 | 0.0101 | 0.0003 | 0.3065 | 58 | 5 | 65 | 2 |
| CSS-008-7* | 0.83 | 0.0791 | 0.0038 | 0.1067 | 0.0051 | 0.0098 | 0.0002 | 0.5335 | 59 | 7 | 60 | 2 |
| CSS-008-10 | 0.74 | 0.0441 | 0.0039 | 0.0594 | 0.0052 | 0.0098 | 0.0003 | 0.2930 | 59 | 5 | 63 | 2 |
| CSS-008-11 | 0.93 | 0.0590 | 0.0033 | 0.0818 | 0.0046 | 0.0101 | 0.0003 | 0.4400 | 80 | 4 | 65 | 2 |
| CSS-008-12 | 1.61 | 0.0436 | 0.0017 | 0.0574 | 0.0024 | 0.0096 | 0.0002 | 0.5856 | 57 | 2 | 61 | 1 |
| CSS-008-13 | 1.91 | 0.0474 | 0.0018 | 0.0632 | 0.0025 | 0.0097 | 0.0002 | 0.6318 | 62 | 2 | 62 | 2 |
| CSS-008-14 | 1.06 | 0.0690 | 0.0036 | 0.0940 | 0.0049 | 0.0099 | 0.0002 | 0.4828 | 60 | 7 | 61 | 2 |
| CSS-008-15 | 1.39 | 0.0593 | 0.0024 | 0.0797 | 0.0033 | 0.0098 | 0.0002 | 0.5963 | 60 | 4 | 62 | 2 |
| CSS-008-16 | 1.01 | 0.0458 | 0.0034 | 0.0603 | 0.0046 | 0.0095 | 0.0002 | 0.3332 | 59 | 4 | 61 | 2 |
| CSS-008-17 | 1.10 | 0.0486 | 0.0029 | 0.0639 | 0.0038 | 0.0095 | 0.0002 | 0.4228 | 63 | 4 | 61 | 2 |
| CSS-008-18 | 1.09 | 0.0495 | 0.0023 | 0.0675 | 0.0032 | 0.0099 | 0.0003 | 0.5284 | 66 | 3 | 63 | 2 |
| CSS-008-19* | 1.11 | 0.1465 | 0.0053 | 0.2290 | 0.0084 | 0.0113 | 0.0003 | 0.7213 | 78 | 19 | 64 | 2 |
| CSS-008-20 | 0.97 | 0.0666 | 0.0030 | 0.0924 | 0.0043 | 0.0101 | 0.0003 | 0.5338 | 90 | 4 | 65 | 2 |
| CSS-008-21 | 0.90 | 0.0469 | 0.0031 | 0.0629 | 0.0042 | 0.0097 | 0.0003 | 0.3832 | 62 | 4 | 62 | 2 |
| CSS-008-22 | 1.10 | 0.0452 | 0.0033 | 0.0600 | 0.0044 | 0.0096 | 0.0003 | 0.3560 | 59 | 4 | 62 | 2 |
| CSS-008-23 | 0.97 | 0.0465 | 0.0024 | 0.0630 | 0.0033 | 0.0098 | 0.0002 | 0.4608 | 62 | 3 | 63 | 2 |
| CSS-008-25 | 1.23 | 0.0349 | 0.0029 | 0.0470 | 0.0039 | 0.0098 | 0.0003 | 0.3118 | 47 | 4 | 63 | 2 |
| CSS-008-26 | 0.87 | 0.0483 | 0.0032 | 0.0644 | 0.0043 | 0.0097 | 0.0003 | 0.3873 | 63 | 4 | 62 | 2 |
| CSS-008-27 | 1.25 | 0.0425 | 0.0017 | 0.0576 | 0.0023 | 0.0098 | 0.0002 | 0.6040 | 57 | 2 | 63 | 2 |
| CSS-008-28* | 0.94 | 0.1366 | 0.0042 | 0.2067 | 0.0067 | 0.0110 | 0.0003 | 0.7564 | 61 | 13 | 62 | 2 |
| CSS-008-29 | 0.82 | 0.0443 | 0.0026 | 0.0600 | 0.0036 | 0.0098 | 0.0002 | 0.4261 | 59 | 3 | 63 | 2 |
| ***Not used*** |  |  |  |  |  |  |  |  |  |  |  |  |
| CSS-008-4* | 1.41 | 0.0465 | 0.0030 | 0.0593 | 0.0039 | 0.0093 | 0.0002 | 0.3822 | 59 | 4 | 59 | 1 |
| CSS-008-8* | 1.21 | 0.0572 | 0.0033 | 0.0747 | 0.0043 | 0.0095 | 0.0002 | 0.4382 | 58 | 3 | 60 | 1 |
|  |  |  |  |  |  |  |  |  |  |  |  |  |
| ***CSS-009 Fayalite-absent syenite*** | | |  |  |  |  |  |  |  |  |  |  |
| CSS-009-1 | 1.44 | 0.0416 | 0.0018 | 0.0554 | 0.0025 | 0.0096 | 0.0002 | 0.5258 | 55 | 2 | 62 | 1 |
| CSS-009-2 | 1.25 | 0.0471 | 0.0024 | 0.0623 | 0.0033 | 0.0096 | 0.0002 | 0.4599 | 61 | 3 | 61 | 1 |
| CSS-009-3 | 0.69 | 0.0521 | 0.0043 | 0.0690 | 0.0057 | 0.0096 | 0.0002 | 0.3174 | 68 | 5 | 62 | 2 |
| CSS-009-4* | 0.61 | 0.1745 | 0.0058 | 0.2818 | 0.0096 | 0.0117 | 0.0003 | 0.7520 | 65 | 14 | 63 | 2 |
| CSS-009-6 | 1.38 | 0.0465 | 0.0023 | 0.0615 | 0.0031 | 0.0096 | 0.0002 | 0.5047 | 61 | 3 | 61 | 2 |
| CSS-009-7 | 0.87 | 0.0453 | 0.0028 | 0.0620 | 0.0039 | 0.0099 | 0.0003 | 0.4015 | 61 | 4 | 64 | 2 |
| CSS-009-8 | 0.83 | 0.0525 | 0.0030 | 0.0729 | 0.0042 | 0.0101 | 0.0003 | 0.4286 | 71 | 4 | 65 | 2 |
| CSS-009-9 | 0.92 | 0.0607 | 0.0028 | 0.0847 | 0.0040 | 0.0101 | 0.0003 | 0.5190 | 83 | 4 | 65 | 2 |
| CSS-009-10 | 0.76 | 0.0503 | 0.0031 | 0.0676 | 0.0042 | 0.0098 | 0.0002 | 0.4107 | 66 | 4 | 63 | 2 |
| CSS-009-11 | 0.78 | 0.0460 | 0.0028 | 0.0628 | 0.0039 | 0.0099 | 0.0002 | 0.4020 | 62 | 4 | 64 | 2 |
| ***Not used*** |  |  |  |  |  |  |  |  |  |  |  |  |
| CSS-009-5* | 0.62 | 0.4269 | 0.0101 | 1.234 | 0.0322 | 0.0210 | 0.0005 | 0.9322 | 76 | 56 | 70 | 4 |

*Not used in the Concordia plots. All data rounded to four decimal places. Error correlation calculated using five decimal places and rounded to four.

**Supplementary Dataset S2**

**Dataset S1.** Zircon LA-ICP-MS geochronology results from North Island

| *Analysis* | *Th/U* | *^207^Pb/^206^Pb* | ± 1s | *^207^Pb/^235^U* | ± 1s | *^206^Pb/^238^U* | ± 1s | *Error Correlation* | *^207^Pb/^235^U Age (Ma)* | ± 1s | *^206^Pb/^238^U*  *Age (Ma)* | ± 1s |
| --- | --- | --- | --- | --- | --- | --- | --- | --- | --- | --- | --- | --- |
| ***NI-002 Diorite*** |  |  |  |  |  |  |  |  |  |  |  |  |
| NI002-1 | 1.01 | 0.0470 | 0.0015 | 0.0614 | 0.0020 | 0.0095 | 0.0002 | 0.7342 | 61 | 2 | 61 | 1 |
| NI002-2 | 1.05 | 0.0485 | 0.0021 | 0.0656 | 0.0028 | 0.0098 | 0.0003 | 0.5911 | 64 | 3 | 63 | 2 |
| NI002-3 | 0.36 | 0.0483 | 0.0026 | 0.0644 | 0.0035 | 0.0097 | 0.0003 | 0.4741 | 63 | 3 | 62 | 2 |
| NI002-4 | 0.94 | 0.0464 | 0.0020 | 0.0612 | 0.0027 | 0.0096 | 0.0002 | 0.5707 | 60 | 3 | 61 | 2 |
| NI002-5 | 1.29 | 0.0451 | 0.0017 | 0.0597 | 0.0023 | 0.0096 | 0.0002 | 0.6634 | 59 | 2 | 62 | 2 |
| NI002-6 | 0.99 | 0.0476 | 0.0017 | 0.0620 | 0.0023 | 0.0095 | 0.0002 | 0.6672 | 61 | 2 | 61 | 1 |
| NI002-7 | 0.97 | 0.0472 | 0.0019 | 0.0625 | 0.0025 | 0.0096 | 0.0002 | 0.6193 | 62 | 2 | 62 | 2 |
| NI002-8 | 1.13 | 0.0465 | 0.0012 | 0.0621 | 0.0018 | 0.0097 | 0.0002 | 0.8229 | 61 | 2 | 62 | 1 |
| NI002-9 | 1.40 | 0.0571 | 0.0021 | 0.0730 | 0.0027 | 0.0093 | 0.0002 | 0.6678 | 58 | 7 | 59 | 1 |
| NI002-10 | 0.75 | 0.0460 | 0.0016 | 0.0601 | 0.0021 | 0.0095 | 0.0002 | 0.6854 | 59 | 2 | 61 | 1 |
| NI002-11 | 0.78 | 0.0469 | 0.0022 | 0.0625 | 0.0029 | 0.0097 | 0.0003 | 0.5575 | 62 | 3 | 62 | 2 |
| NI002-12 | 0.62 | 0.0492 | 0.0028 | 0.0633 | 0.0036 | 0.0093 | 0.0003 | 0.4712 | 62 | 3 | 60 | 2 |
| NI002-14 | 1.20 | 0.0455 | 0.0015 | 0.0590 | 0.0021 | 0.0094 | 0.0002 | 0.7005 | 58 | 2 | 60 | 1 |
| NI002-15 | 0.95 | 0.0511 | 0.0024 | 0.0648 | 0.0030 | 0.0092 | 0.0002 | 0.5613 | 57 | 3 | 59 | 1 |
| NI002-16 | 1.21 | 0.0480 | 0.0018 | 0.0605 | 0.0023 | 0.0091 | 0.0002 | 0.6501 | 60 | 2 | 59 | 1 |
| NI002-17 | 0.71 | 0.0485 | 0.0015 | 0.0641 | 0.0021 | 0.0096 | 0.0002 | 0.7249 | 63 | 2 | 62 | 1 |
| NI002-18 | 1.10 | 0.0482 | 0.0017 | 0.0639 | 0.0023 | 0.0096 | 0.0002 | 0.6851 | 63 | 2 | 62 | 2 |
| NI002-19 | 1.13 | 0.0490 | 0.0018 | 0.0628 | 0.0024 | 0.0093 | 0.0002 | 0.6555 | 62 | 2 | 60 | 1 |
| NI002-20 | 1.29 | 0.0472 | 0.0018 | 0.0630 | 0.0024 | 0.0097 | 0.0002 | 0.6458 | 62 | 2 | 62 | 2 |
|  |  |  |  |  |  |  |  |  |  |  |  |  |
| ***NI-003 Syenite*** |  |  |  |  |  |  |  |  |  |  |  |  |
| NI003-1 | 1.03 | 0.0495 | 0.0024 | 0.0651 | 0.0031 | 0.0095 | 0.0002 | 0.4811 | 64 | 3 | 61 | 1 |
| NI003-2 | 0.61 | 0.0429 | 0.0060 | 0.0560 | 0.0079 | 0.0095 | 0.0002 | 0.1881 | 55 | 8 | 61 | 2 |
| NI003-3 | 1.93 | 0.0527 | 0.0017 | 0.0724 | 0.0025 | 0.0100 | 0.0002 | 0.6821 | 62 | 4 | 63 | 1 |
| NI003-4 | 0.91 | 0.0497 | 0.0029 | 0.0652 | 0.0038 | 0.0095 | 0.0002 | 0.4130 | 64 | 4 | 61 | 1 |
| NI003-5 | 0.86 | 0.0511 | 0.0029 | 0.0664 | 0.0038 | 0.0094 | 0.0002 | 0.4230 | 65 | 4 | 60 | 1 |
| NI003-6 | 1.00 | 0.0432 | 0.0020 | 0.0556 | 0.0026 | 0.0093 | 0.0002 | 0.4963 | 55 | 3 | 60 | 1 |
| NI003-7 | 1.03 | 0.0457 | 0.0021 | 0.0600 | 0.0028 | 0.0095 | 0.0002 | 0.5173 | 59 | 3 | 61 | 1 |
| NI003-8 | 0.87 | 0.0441 | 0.0029 | 0.0570 | 0.0038 | 0.0094 | 0.0002 | 0.3711 | 56 | 4 | 60 | 1 |
| NI003-9 | 1.03 | 0.0456 | 0.0013 | 0.0604 | 0.0018 | 0.0096 | 0.0002 | 0.7478 | 60 | 2 | 62 | 1 |
| NI003-10 | 0.70 | 0.0570 | 0.0053 | 0.0761 | 0.0071 | 0.0097 | 0.0003 | 0.2789 | 61 | 8 | 61 | 2 |
| NI003-11 | 0.87 | 0.0484 | 0.0036 | 0.0631 | 0.0047 | 0.0095 | 0.0002 | 0.3247 | 62 | 5 | 61 | 1 |
| NI003-12 | 0.92 | 0.0450 | 0.0023 | 0.0590 | 0.0030 | 0.0095 | 0.0002 | 0.4710 | 58 | 3 | 61 | 1 |
| NI003-13 | 0.85 | 0.0479 | 0.0028 | 0.0630 | 0.0037 | 0.0095 | 0.0002 | 0.4319 | 62 | 4 | 61 | 2 |
| NI003-15 | 0.91 | 0.0465 | 0.0028 | 0.0614 | 0.0037 | 0.0096 | 0.0002 | 0.4152 | 60 | 4 | 61 | 2 |
| NI003-16 | 0.52 | 0.0454 | 0.0079 | 0.0600 | 0.0105 | 0.0096 | 0.0003 | 0.1674 | 59 | 10 | 62 | 2 |
| NI003-17 | 1.24 | 0.0496 | 0.0013 | 0.0652 | 0.0018 | 0.0095 | 0.0002 | 0.8592 | 59 | 3 | 61 | 1 |
| NI003-18 | 0.77 | 0.0556 | 0.0016 | 0.0733 | 0.0022 | 0.0096 | 0.0002 | 0.8012 | 59 | 4 | 61 | 1 |
| NI003-19 | 1.03 | 0.0447 | 0.0019 | 0.0588 | 0.0026 | 0.0095 | 0.0002 | 0.5518 | 58 | 2 | 61 | 1 |
| NI003-20 | 1.07 | 0.0469 | 0.0024 | 0.0619 | 0.0032 | 0.0096 | 0.0002 | 0.4914 | 61 | 3 | 61 | 2 |
| ***Not used*** |  |  |  |  |  |  |  |  |  |  |  |  |
| NI003-14* | 1.08 | 0.0461 | 0.0020 | 0.0671 | 0.0029 | 0.0106 | 0.0003 | 0.5620 | 66 | 3 | 68 | 2 |
|  |  |  |  |  |  |  |  |  |  |  |  |  |
| ***NI-004 Syenite*** |  |  |  |  |  |  |  |  |  |  |  |  |
| NI004-1 | 0.69 | 0.0565 | 0.0075 | 0.0735 | 0.0098 | 0.0094 | 0.0003 | 0.2237 | 72 | 9 | 61 | 2 |
| NI004-2 | 0.65 | 0.0393 | 0.0082 | 0.0513 | 0.0106 | 0.0095 | 0.0003 | 0.1376 | 51 | 10 | 61 | 2 |
| NI004-3 | 0.71 | 0.0467 | 0.0050 | 0.0635 | 0.0068 | 0.0099 | 0.0003 | 0.2465 | 62 | 6 | 63 | 2 |
| NI004-4 | 0.96 | 0.0454 | 0.0035 | 0.0594 | 0.0046 | 0.0095 | 0.0002 | 0.3246 | 59 | 4 | 61 | 2 |
| NI004-5 | 1.05 | 0.0435 | 0.0035 | 0.0564 | 0.0045 | 0.0094 | 0.0002 | 0.3194 | 56 | 4 | 60 | 2 |
| NI004-6 | 1.94 | 0.0448 | 0.0017 | 0.0588 | 0.0023 | 0.0095 | 0.0002 | 0.6172 | 58 | 2 | 61 | 1 |
| NI004-7 | 1.29 | 0.0464 | 0.0013 | 0.0613 | 0.0019 | 0.0096 | 0.0002 | 0.7864 | 60 | 2 | 62 | 1 |
| NI004-8 | 0.82 | 0.0450 | 0.0046 | 0.0581 | 0.0060 | 0.0094 | 0.0003 | 0.2681 | 57 | 6 | 60 | 2 |
| NI004-9 | 0.68 | 0.0385 | 0.0077 | 0.0530 | 0.0106 | 0.0100 | 0.0003 | 0.1403 | 52 | 10 | 64 | 2 |
| NI004-10 | 0.76 | 0.0374 | 0.0052 | 0.0489 | 0.0068 | 0.0095 | 0.0003 | 0.1892 | 48 | 7 | 61 | 2 |
| NI004-11 | 0.97 | 0.0428 | 0.0038 | 0.0569 | 0.0050 | 0.0097 | 0.0002 | 0.2815 | 56 | 5 | 62 | 2 |
| NI004-12 | 0.71 | 0.0495 | 0.0043 | 0.0652 | 0.0057 | 0.0096 | 0.0003 | 0.2987 | 64 | 5 | 61 | 2 |
| NI004-13 | 0.72 | 0.0353 | 0.0060 | 0.0458 | 0.0078 | 0.0094 | 0.0003 | 0.1570 | 45 | 8 | 60 | 2 |
| NI004-14 | 0.88 | 0.0381 | 0.0034 | 0.0525 | 0.0047 | 0.0100 | 0.0003 | 0.2789 | 52 | 5 | 64 | 2 |
| NI004-15 | 0.79 | 0.0475 | 0.0045 | 0.0606 | 0.0057 | 0.0093 | 0.0002 | 0.2742 | 60 | 5 | 59 | 2 |
| NI004-16 | 0.76 | 0.0340 | 0.0047 | 0.0444 | 0.0061 | 0.0095 | 0.0002 | 0.1830 | 44 | 6 | 61 | 2 |
| NI004-17 | 0.81 | 0.0494 | 0.0044 | 0.0634 | 0.0056 | 0.0093 | 0.0002 | 0.2925 | 62 | 5 | 60 | 2 |
| NI004-18 | 2.28 | 0.0427 | 0.0018 | 0.0551 | 0.0024 | 0.0094 | 0.0002 | 0.5485 | 54 | 2 | 60 | 1 |
| NI004-19 | 0.83 | 0.0514 | 0.0031 | 0.0662 | 0.0041 | 0.0093 | 0.0002 | 0.4018 | 65 | 4 | 60 | 1 |
| NI004-20 | 0.88 | 0.0472 | 0.0031 | 0.0612 | 0.0041 | 0.0094 | 0.0002 | 0.3679 | 60 | 4 | 60 | 1 |
|  |  |  |  |  |  |  |  |  |  |  |  |  |
| **NI-010 Syenite** |  |  |  |  |  |  |  |  |  |  |  |  |
| NI010-1 | 0.62 | 0.0454 | 0.0061 | 0.0594 | 0.0079 | 0.0095 | 0.0003 | 0.2056 | 59 | 8 | 61 | 2 |
| NI010-2 | 0.56 | 0.0325 | 0.0068 | 0.0423 | 0.0088 | 0.0094 | 0.0003 | 0.1369 | 42 | 9 | 61 | 2 |
| NI010-3 | 0.60 | 0.0474 | 0.0073 | 0.0599 | 0.0093 | 0.0092 | 0.0003 | 0.1908 | 59 | 9 | 59 | 2 |
| NI010-4 | 0.35 | 0.0563 | 0.0085 | 0.0730 | 0.0110 | 0.0094 | 0.0003 | 0.1981 | 72 | 10 | 60 | 2 |
| NI010-5 | 0.38 | 0.0398 | 0.0068 | 0.0507 | 0.0087 | 0.0092 | 0.0003 | 0.1645 | 50 | 8 | 59 | 2 |
| NI010-6 | 0.76 | 0.0405 | 0.0039 | 0.0525 | 0.0051 | 0.0094 | 0.0003 | 0.2748 | 52 | 5 | 60 | 2 |
| NI010-7 | 0.54 | 0.0605 | 0.0106 | 0.0767 | 0.0134 | 0.0092 | 0.0003 | 0.1808 | 75 | 13 | 59 | 2 |
| NI010-8 | 0.60 | 0.0375 | 0.0078 | 0.0490 | 0.0101 | 0.0095 | 0.0003 | 0.1428 | 49 | 10 | 61 | 2 |
| NI010-9 | 0.52 | 0.0438 | 0.0092 | 0.0565 | 0.0118 | 0.0094 | 0.0003 | 0.1481 | 56 | 11 | 60 | 2 |
| NI010-10 | 1.17 | 0.0469 | 0.0014 | 0.0627 | 0.0020 | 0.0097 | 0.0002 | 0.7548 | 62 | 2 | 62 | 1 |
| NI010-11 | 0.35 | 0.0545 | 0.0093 | 0.0716 | 0.0122 | 0.0095 | 0.0003 | 0.1854 | 70 | 12 | 61 | 2 |
| NI010-12 | 0.38 | 0.0380 | 0.0098 | 0.0502 | 0.0129 | 0.0096 | 0.0003 | 0.1218 | 50 | 12 | 61 | 2 |
| NI010-13 | 0.49 | 0.0470 | 0.0054 | 0.0633 | 0.0073 | 0.0098 | 0.0003 | 0.2399 | 62 | 7 | 63 | 2 |
| NI010-14 | 0.35 | 0.0423 | 0.0105 | 0.0563 | 0.0139 | 0.0096 | 0.0003 | 0.1344 | 56 | 13 | 62 | 2 |
| NI010-15 | 0.41 | 0.0430 | 0.0057 | 0.0583 | 0.0077 | 0.0098 | 0.0003 | 0.2147 | 58 | 7 | 63 | 2 |
| NI010-16 | 0.51 | 0.0312 | 0.0078 | 0.0387 | 0.0097 | 0.0090 | 0.0003 | 0.1245 | 39 | 9 | 58 | 2 |
| NI010-17 | 0.56 | 0.0230 | 0.0069 | 0.0302 | 0.0091 | 0.0095 | 0.0003 | 0.0979 | 30 | 9 | 61 | 2 |
| NI010-18 | 0.59 | 0.0397 | 0.0077 | 0.0534 | 0.0103 | 0.0097 | 0.0003 | 0.1538 | 53 | 10 | 62 | 2 |
| NI010-19 | 0.52 | 0.0353 | 0.0071 | 0.0471 | 0.0095 | 0.0097 | 0.0003 | 0.1440 | 47 | 9 | 62 | 2 |
| NI010-20 | 0.37 | 0.0397 | 0.0077 | 0.0535 | 0.0104 | 0.0098 | 0.0003 | 0.1522 | 53 | 10 | 63 | 2 |
|  |  |  |  |  |  |  |  |  |  |  |  |  |
| ***NI-017 Syenite*** |  |  |  |  |  |  |  |  |  |  |  |  |
| NI017-1 | 0.65 | 0.0535 | 0.0026 | 0.0702 | 0.0034 | 0.0095 | 0.0003 | 0.5355 | 59 | 3 | 61 | 1 |
| NI017-2 | 0.60 | 0.0497 | 0.0021 | 0.0647 | 0.0028 | 0.0094 | 0.0002 | 0.5981 | 64 | 3 | 61 | 2 |
| NI017-3 | 0.44 | 0.0475 | 0.0025 | 0.0620 | 0.0032 | 0.0095 | 0.0002 | 0.4880 | 61 | 3 | 61 | 2 |
| NI017-4 | 0.50 | 0.0507 | 0.0032 | 0.0651 | 0.0041 | 0.0093 | 0.0003 | 0.4287 | 64 | 4 | 60 | 2 |
| NI017-5 | 0.54 | 0.0520 | 0.0028 | 0.0668 | 0.0036 | 0.0093 | 0.0002 | 0.4837 | 58 | 3 | 59 | 1 |
| NI017-6 | 0.69 | 0.0467 | 0.0021 | 0.0616 | 0.0029 | 0.0096 | 0.0002 | 0.5401 | 61 | 3 | 61 | 2 |
| NI017-7 | 0.43 | 0.0569 | 0.0057 | 0.0739 | 0.0073 | 0.0094 | 0.0003 | 0.3231 | 72 | 7 | 60 | 2 |
| NI017-8 | 0.57 | 0.0456 | 0.0026 | 0.0600 | 0.0035 | 0.0096 | 0.0003 | 0.4525 | 59 | 3 | 61 | 2 |
| NI017-9 | 1.06 | 0.0471 | 0.0015 | 0.0625 | 0.0021 | 0.0096 | 0.0002 | 0.7412 | 62 | 2 | 62 | 2 |
| NI017-10 | 1.03 | 0.0495 | 0.0021 | 0.0661 | 0.0028 | 0.0097 | 0.0003 | 0.6056 | 65 | 3 | 62 | 2 |
| NI017-11 | 0.53 | 0.0502 | 0.0034 | 0.0643 | 0.0044 | 0.0093 | 0.0003 | 0.3972 | 63 | 4 | 60 | 2 |
| NI017-12 | 0.71 | 0.0524 | 0.0027 | 0.0673 | 0.0035 | 0.0093 | 0.0002 | 0.4979 | 66 | 3 | 60 | 2 |
| NI017-13 | 0.64 | 0.0483 | 0.0025 | 0.0619 | 0.0032 | 0.0093 | 0.0002 | 0.4969 | 61 | 3 | 60 | 2 |
| NI017-14 | 0.72 | 0.0478 | 0.0019 | 0.0634 | 0.0026 | 0.0096 | 0.0002 | 0.6201 | 62 | 2 | 62 | 2 |
| NI017-15 | 0.64 | 0.0472 | 0.0032 | 0.0633 | 0.0042 | 0.0097 | 0.0003 | 0.4009 | 62 | 4 | 62 | 2 |
| NI017-16 | 0.75 | 0.0470 | 0.0024 | 0.0624 | 0.0033 | 0.0096 | 0.0003 | 0.4970 | 61 | 3 | 62 | 2 |
| NI017-17 | 0.99 | 0.0604 | 0.0023 | 0.0812 | 0.0031 | 0.0098 | 0.0003 | 0.6648 | 79 | 3 | 63 | 2 |
| NI017-18 | 0.53 | 0.0451 | 0.0045 | 0.0575 | 0.0056 | 0.0092 | 0.0003 | 0.2991 | 57 | 5 | 59 | 2 |
| NI017-19 | 0.50 | 0.0479 | 0.0037 | 0.0632 | 0.0049 | 0.0096 | 0.0003 | 0.3663 | 62 | 5 | 61 | 2 |
| NI017-20 | 0.62 | 0.0477 | 0.0026 | 0.0628 | 0.0034 | 0.0096 | 0.0003 | 0.4837 | 62 | 3 | 61 | 2 |

*Not used in the Concordia plots. All data rounded to four decimal places. Error correlation calculated using five decimal places and rounded to four.


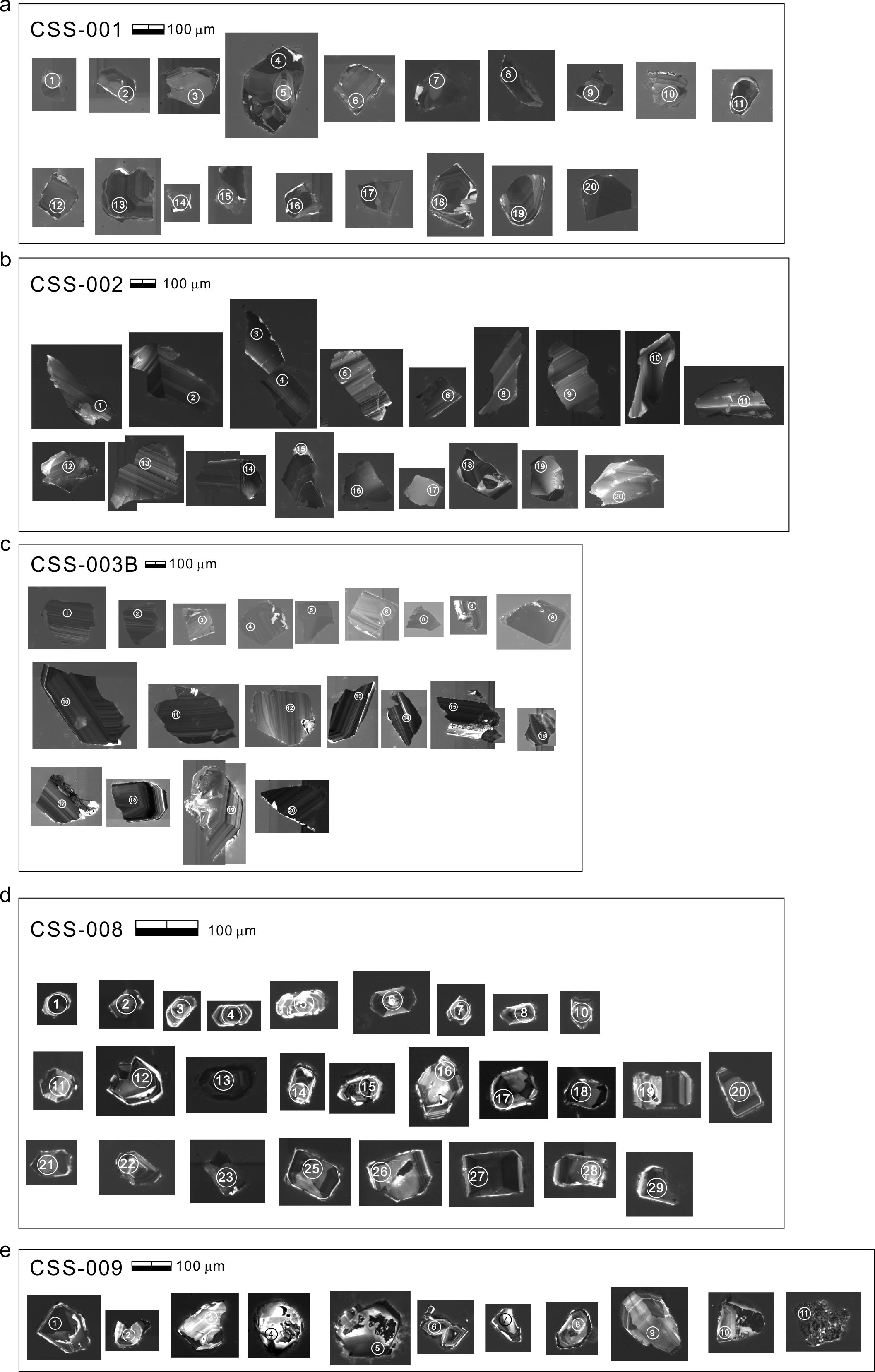


**Figure S1. Cathodoluminescence images of the zircons from Silhouette rocks analyzed for *in situ* U/Pb geochronology**. The number corresponds to the spot location and number. (**a**) CSS-001 (fayalite syenite), (**b**) CSS-002 (fayalite syenite), (**c**) CSS-003 (fayalite syenite), (**d**) CSS-008 (microrgranite), (**e**) CSS-009 (syenite).


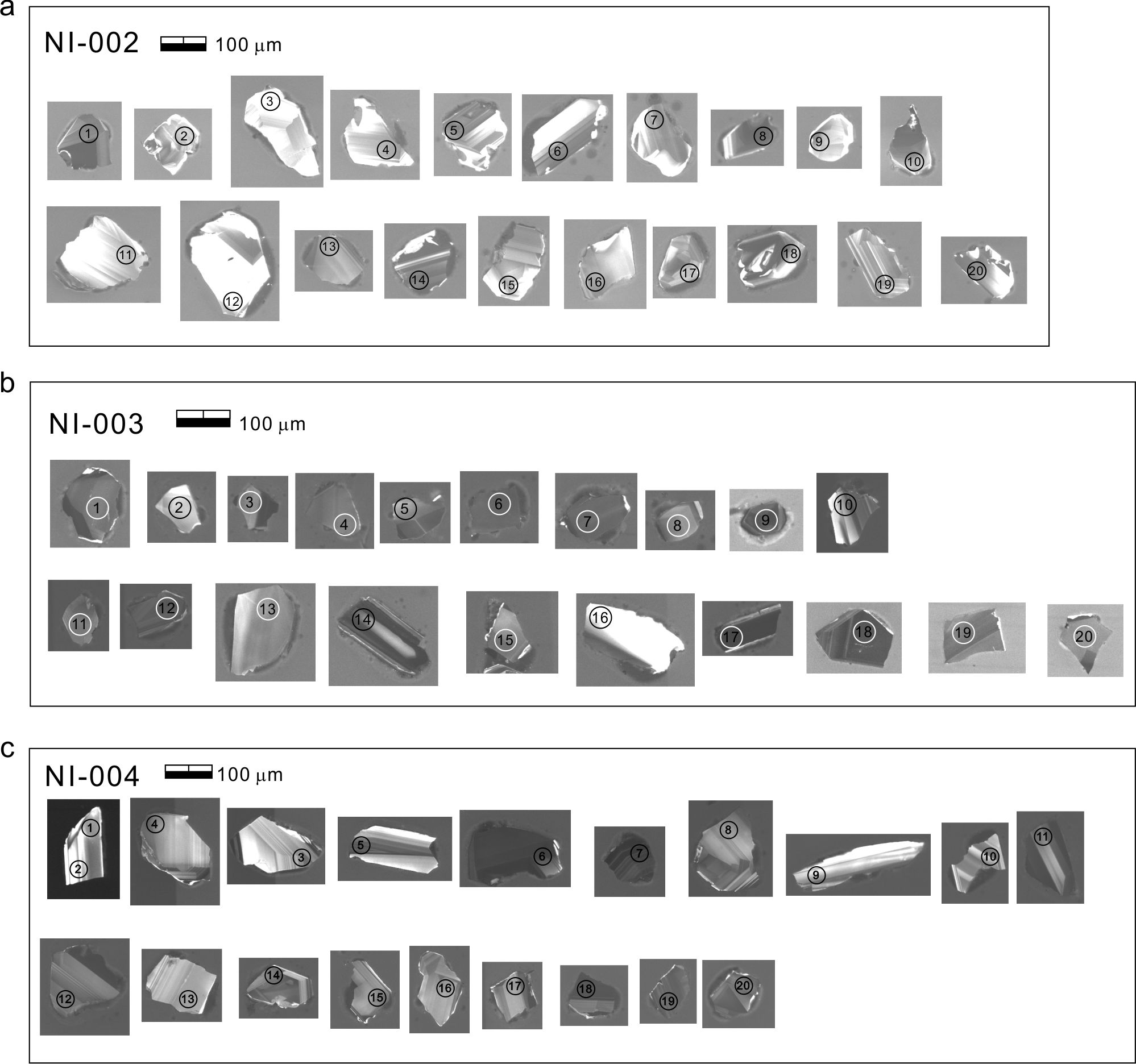


**
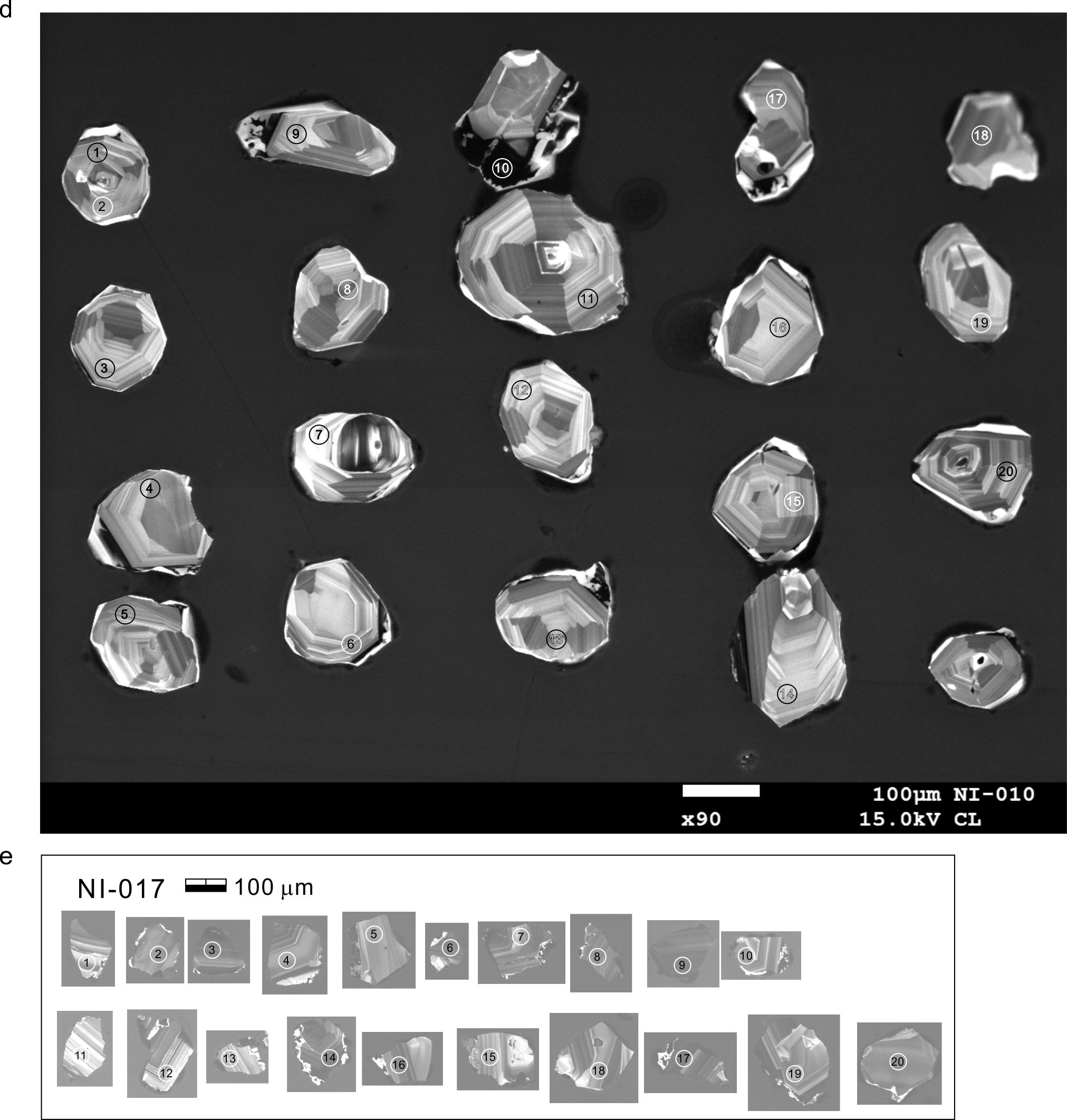
**

**Figure S2. Cathodoluminescence images of the zircons from North Island rocks analyzed for *in situ* U/Pb geochronology**. The number corresponds to the spot location and number. (**a**) NI-002 (diorite), (**b**) NI-003 (syenite), (**c**) NI-004 (syenite), (**d**) NI-010 (syenite), (**e**) NI-017 (syenite).


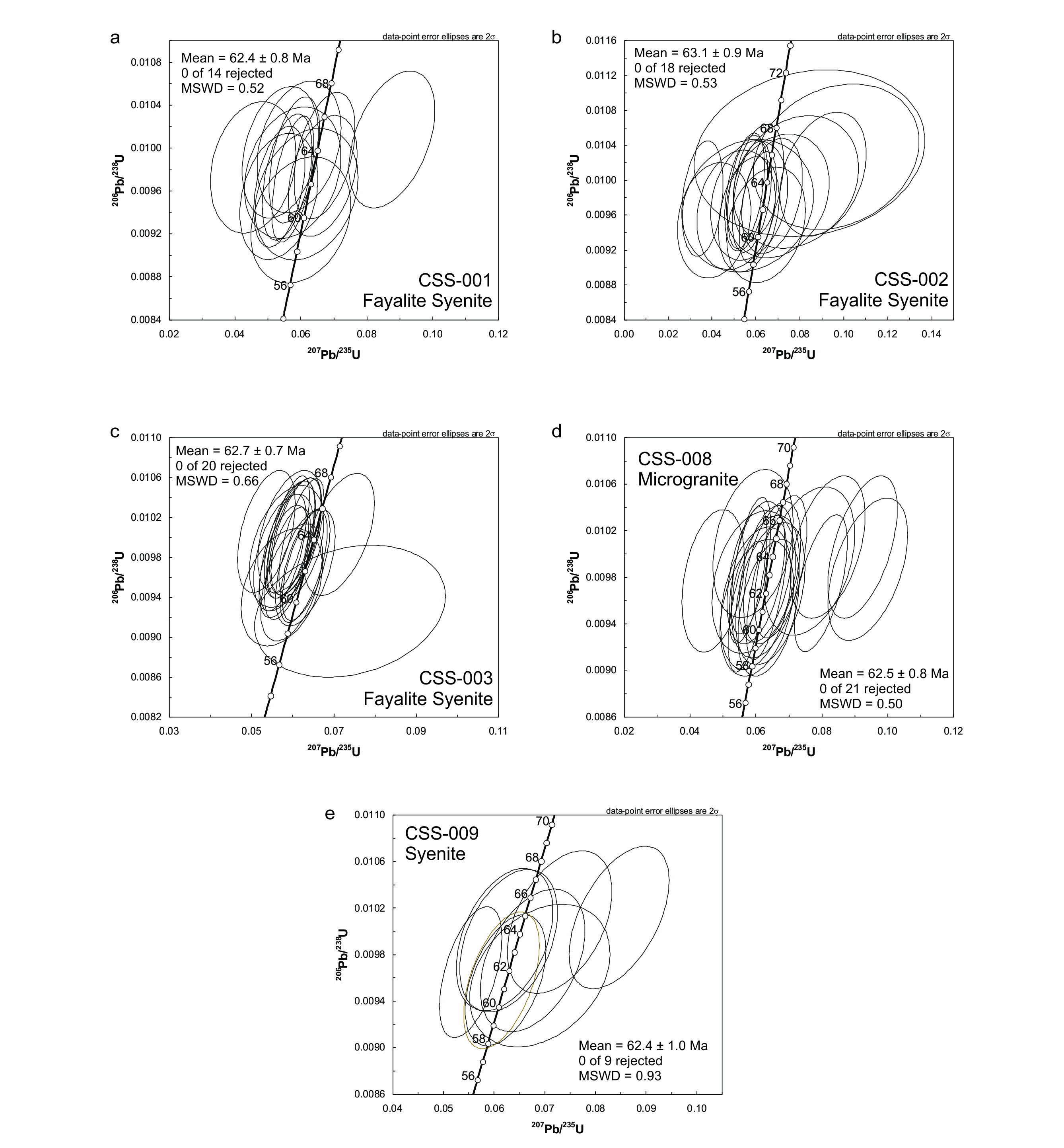


**Figure S3**. **Concordia diagrams of the rocks from Silhouette with the weighted mean ages**. (**a**) CSS-001 (fayalite syenite), (**b**) CSS-002 (fayalite syenite), (**c**) CSS-003 (fayalite syenite), (**d**) CSS-008 (microrgranite), (**e**) CSS-009 (syenite).


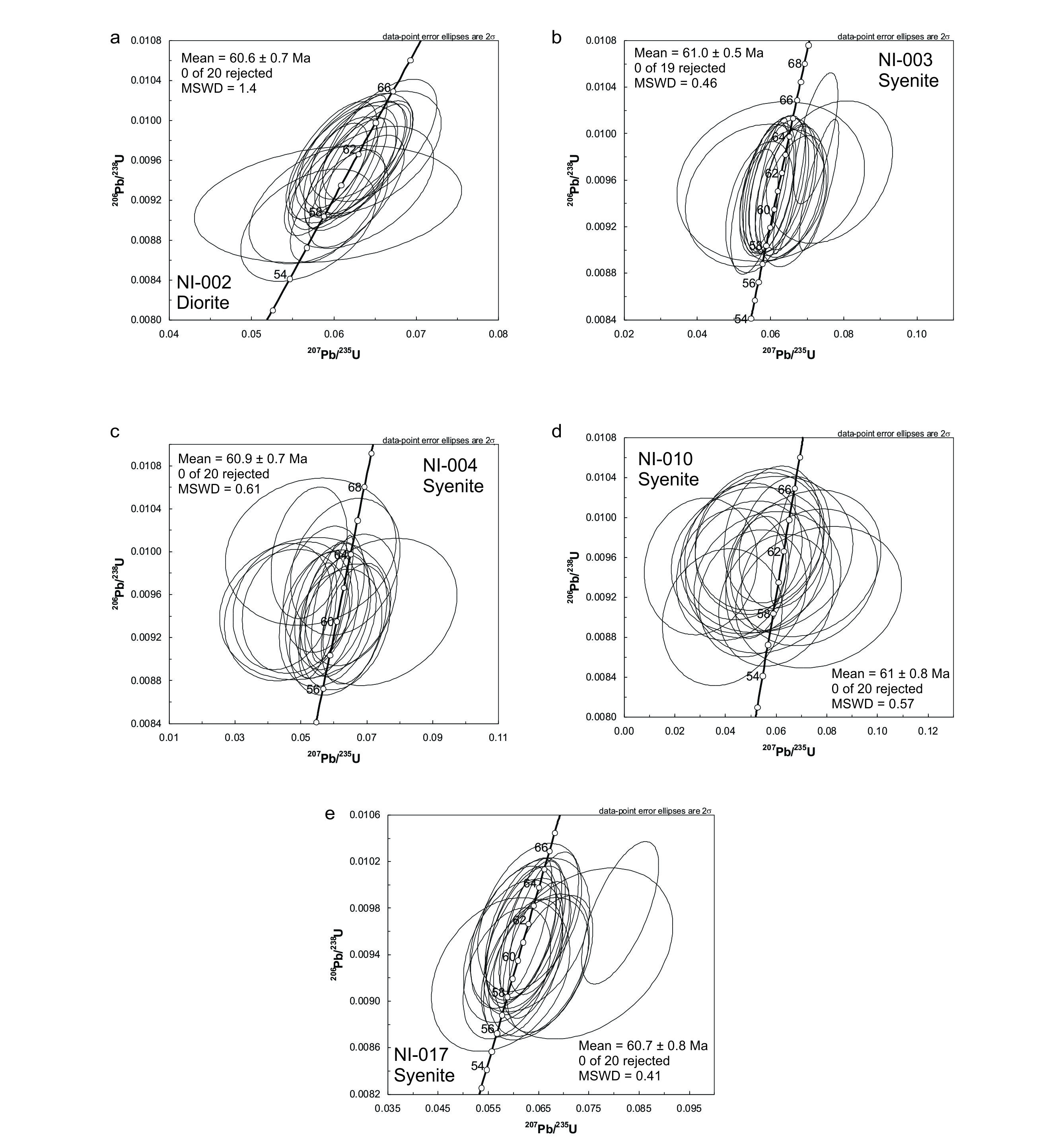


**Figure S4**. **Concordia diagrams of the rocks from North Island with the weighted mean ages**. (**a**) NI-002 (diorite), (**b**) NI-003 (syenite), (**c**) NI-004 (syenite), (**d**) NI-010 (syenite), (**e**) NI-017 (syenite).
